# Time-domain diffuse correlation spectroscopy at large source detector separation for cerebral blood flow recovery

**DOI:** 10.1364/BOE.523514

**Published:** 2024-06-26

**Authors:** Neda Mogharari, Stanisław Wojtkiewicz, Dawid Borycki, Adam Liebert, Michał Kacprzak

**Affiliations:** 1 Nalecz Institute of Biocybernetics and Biomedical Engineering, Polish Academy of Sciences, Poland; 2 Institute of Physical Chemistry, Polish Academy of Sciences, Kasprzaka 44/52, Poland

## Abstract

Time-domain diffuse correlation spectroscopy (td-DCS) enables the depth discrimination in tissue’s blood flow recovery, considering the fraction of photons detected with higher time of flight (TOF) and longer pathlength through the tissue. However, the recovery result depends on factors such as the instrument response function (IRF), analyzed TOF gate start time, gate width and the source-detector separation (SDS). In this research we evaluate the performance of the td-DCS technique at three SDSs of 1.5, 2 and 2.5 cm to recover cerebral blood flow (CBF). To do that we presented comprehensive characterization of the td-DCS system through a series of phantom experiments. First by quality metrices such as coefficient of variation and contrast-to-noise ratios, we identified optimal time gate(s) of the TOF to extract dynamics of particles. Then using sensitivity metrices, each SDS ability to detect dynamics of particles in superficial and deeper layer was evaluated. Finally, td-DCS at each SDS was tested on healthy volunteers during cuff occlusion test and breathing tasks. According to phantom measurements, the sensitivity to estimate perfusion within the deep layer located at depth of 1.5 cm from the surface can be increased more than two times when the SDS increases from 1.5 cm to 2.5 cm.

## 1. Introduction

CBF provides the needed oxygen to maintain the brain functional activity and structural integrity. An interruption in CBF can cause brain dysfunction, and if the interruption persists, it may lead to the brain death. While the CBF is not routinely measured because of the lack of convenient and reliable technologies, monitoring of CBF is essential in neurosurgery and neurology [1].

Diffuse correlation spectroscopy (DCS) is an optical technique that estimates blood flow index (BFI) by measuring the temporal autocorrelation function of the light intensity fluctuations diffusely reflected from the tissue. In this method, photons emerging from a scattering medium are diffused with different phases that cause strong spatial variations of intensity, or the so-called speckle pattern [2]. If scattering elements move, the speckle intensity fluctuates over time due to slight changes in photon path [3]. In brain imaging and neuro monitoring, the hemodynamic in deep tissues (such as the cerebral hemodynamic) is more valuable and informative for the diagnosis and treatment of diseases than the hemodynamic of superficial tissues. In the continuous wave DCS (cw-DCS) method, discrimination the propagation path of detected photons is not feasible using a single source-detector pair [4] and the recovered blood flow is spatially integrated over all photon paths. As a result of the diffuse nature of the light transport in tissues, the probability of detecting photons with longer pathlength (coming from deep layers) is higher at large SDS [5–7]. In the td-DCS technique which a pulsed laser is used to illuminate the tissue, photons’ paths can be differentiated based on the measured distribution of time of flight of photons (DTOF) associated with varying paths travelled in tissue [8]. td-DCS technique is usually applied in the null-distance or short SDS [6,9–12]. In this method, the sensitivity to the BFI in deep tissues can be improved by analysing photons with higher time of flights (longer pathlengths) which interrogate deeper layers (brain). Although parameters including limited coherence length of lasers used in td-DCS and IRF width makes this method challenging. While lasers with narrow IRF are preferable for time resolved systems, a narrower IRF means a lower coherence length of laser. So, photons in late time gates have less coherency which leads to an increase in the error of estimating the dynamics of particles [13,14].

It has been reported that analysing the photons within the time range around the DTOF peak improves the signal to noise ratio for BFI estimation [9,10,12]. In time domain near infrared (td-NIRS) spectroscopy an increase in SDS, shift the DTOF peak to later time of flight and broadens the DTOF [15]. In this study we investigate the impact of increasing SDS in the td-DCS depth sensitivity to recover CBF. As in DCS technique single-mode fiber with micrometres core diameter and low numerical aperture is used to collect photons, it leads to decrease the number of detected photons at larger SDS. While observing the human tissue safety criteria of ANSI Z136.3 (2018), we increased the illumination area and laser power to apply the td-DCS at large SDSs. This strategy has been successfully applied in td-NIRS [16] and the td-DCS during Valsalva manure [17] and arm occlusion test [18].

The td-DCS technique is known for its relatively poor time gating capabilities, a limitation that has been well-documented in existing literature [19]. Due to this inherent drawback, there is a significant need to validate the performance of td-DCS under conditions of long SDS and using various time gates. Exploring these parameters, we aim to understand better the applicability of the td-DCS, potentially leading to improvements in the technique’s accuracy and stratifying deeper tissue dynamics.

The testing strategy starts with characterization of the td-DCS system at SDSs of 1.5, 2 and 2.5 cm to find the optimal time ranges of the measured DTOFs based on proposed measurands and metrices. Further, the sensitivity metrices of the td-DCS at each SDSs were evaluated on two-layer phantoms with varying dynamics and layers’ thicknesses. Finally, in vivo experiments were carried out on the forearm and forehead of healthy volunteers to compare different SDS’s ability to detect stratified depth blood flow changes in muscles during the response to the occlusion test and in brain cortex during breathing tasks.

## 2. Theory

In the DCS technique, detected photons are time-tagged with the time-correlated single-photon counting (TCSPC) electronics. The TCSPC module measures two parameters for each detected photon: time of flight *t* and the absolute arrival time of photons *t*_τ_. The first tag (picoseconds scale) refers to the time that photons travel from the source to the detector and is used to build the DTOF. The second tag corresponds to the absolute arrival time since the start of measurement (data collection) and is employed to calculate the intensity autocorrelation function $g_{2}(t,\tau )$ , defined as [20]:

(1) $$g_{2}(t,\tau )=\frac{\langle I(t,t_{\tau })I(t,t_{\tau }+\tau )\rangle }{\langle I{\left(t,t_{\tau })\right\rangle }^{2}},$$

where *t* is the time of flight (TOF) and has been calculated considering the zero time at the 1% of the IRF peak on the rising edge, τ is the autocorrelation delay time, *t*_τ_ is the data collection time, *I*(*t*,*t*_τ_) is the light intensity (detected number of photons) that travelled *t* pico seconds between the source and detector and arrived *t*_τ_ seconds since the data collection started. ⟨…⟩ operator stands for the temporal averaging. The $g_{2}(t,\tau )$ can be linked to the normalized electric field autocorrelation function $g_{1}(t,\tau )$ through the Siegert relation [21,22]:

(2) $$g_{2}(t,\tau )=1+\beta |g_{1}(t,\tau ){|}^{2},$$

where *β* is the system parameter (the intercept of $g_{2}$ at *τ*→0) which depends on the laser coherence length and number of modes in the detection fiber [8] and in case of the td-DCS also on the width of the analysed time gate. The $g_{1}(t,\;\tau )$ at a given time gate spanning between *t*_1_ and *t*_2_ is modelled by the following equation [14,23]:

(3) $$g_{1}(t,\tau )=\int_{t_{1}}^{t_{2}}P(t)g_{1}(t,\tau )\text{d}t,$$

and considering a semi-infinite tissue model:

(4) $$g_{1}(t,\tau )=\text{exp}(-k\alpha D_{\text{B}}t\tau ),$$

where $k=2\mu_{\text{s}}^{\prime }k_{0}^{2}{\text{c}}_{0}/n$ , $k_{0}$ is the wavenumber of the light in the medium, *λ* is the light wavelength, *n* is the tissue refractive index, c_0_ refers to the speed of light and *μ*′_s_ is the tissue reduced scattering coefficient. Here the P(t) is the temporal point spread function (e.g. the calculated/simulated distribution of time of flight of photons) in the medium. The BFI equals the *αD*_B_ term, where the *α* refers to the fraction of dynamic to the total scattering events (ranging from 0 to 1), and $D_{\text{B}}$ is the particle diffusion coefficient of the moving particles [3,8,24].

## 3. Methods

### 3.1 td-DCS system

**Fig. 1.**
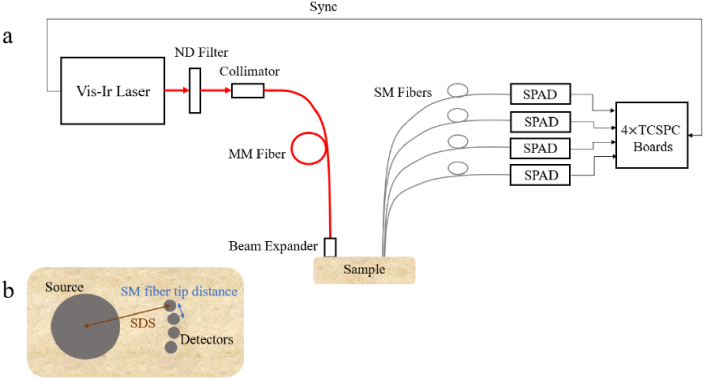
(a) Schematics of the td-DCS system. ND filter: natural density filter, MM fiber: multi-mode fiber, SM fiber: single-mode fiber, SPAD: single-photon avalanche photodiodes, TCSPC: time-correlated single-photon counting. (b) The top view of the sample showing the fibers geometry: SDS – source-detector separation, fibers are not to scale. The SM fibers geometry limits the SM fiber tip distance to 3.5 mm.

The experimental setup as shown in Fig. 1(a) is equipped with a pulsed laser (VisIR-765-HP “STED”, PicoQuant) with 80 Mhz repetition rate at the wavelength of 765 nm. The laser coherence length is 3.5 cm and its IRF is 570 ps in width at the FWHM. At the detection side, 4 single photon avalanche diode (SPAD) (PDM, Micro Photon Devices) and 4 time- correlated single-photon counting (TCSPC) electronic (SPC-150, Becker&Hickl) are synchronized with the pulsed laser.

The TCSPC provides 3.5 ps temporal resolution of the DTOF and 12.5 ns for calculation of the intensity autocorrelation. The laser light is coupled to the multi-mode (MM) fiber with a collimator. To prevent TCSPC boards overflow, the photon count rate was adjusted to be around 170⋅000 counts per second. Therefore, neutral density (ND) filters were used at the laser output. Each SDS requires a separate ND filter. The MM source fiber has 600 µm core diameter and 0.39 numerical aperture. The diffusively reflected light is collected with four single-mode (SM) fibers (780 HP, Thorlabs, USA) of 4.4 µm core diameter. The IRF was measured by positioning the source and detection fibers tip in front of each other. A sheet of white paper covered the detection fiber tip to fill up the whole numerical aperture of the fibers. To apply a high laser power, a beam expander was used to enlarge the illumination area to 13 mm in diameter. The beam expander allowed to be in line with the safety regulations as the power density should not exceed 2.7 mW/mm^2^. So, the system delivers power density of 1.9 mW/mm^2^ at the sample surface. To improve the signal to noise ratio, the data (*g*_2_) from all four SPADs were averaged at each SDS separately. As shown in Fig. 1(b), the SDS is measured from the centre of the illumination area to the centre of the SM fiber tip. All four SM fibers were located at the same SDS. The distance between the SM fibers is 3.5 mm which is the shortest possible distance due to fibre’s geometry.

### 3.2 Physical phantom

**Fig. 2.**
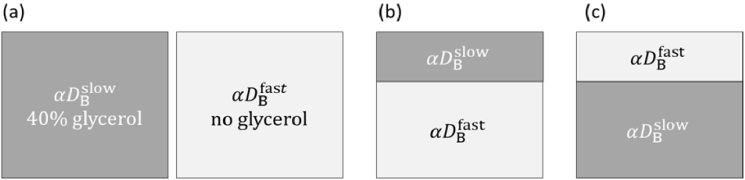
Homogenous and two-layer phantoms measurement scheme: (a) separate homogenous phantoms of flow in superficial and deep layers where $\alpha D_{\text{B}}^{\text{fast}}>\alpha D_{\text{B}}^{\text{slow}}$ , (b) two-layer phantom with higher flow in deep layer and (c) the opposite, where the flow is higher in the superficial layer.

The phantom experiments were conducted in three steps using homogeneous and two-layer phantoms, as outlined in Fig. 2.

We have utilized one-layer and two-layer containers 3D printed of black ABS material. The two-layer container was designed with various superficial layer thickness. The layers are separated by a transparent thin film (maylar with the thickness of 23 µm). The thickness of superficial layer was set to 0.5 cm, 1 cm and 1.5 cm according to the typical scalp and skull thickness of an adult head following [25,26]. Each step of the experiments was conducted separately, at SDSs of 1.5, 2, and 2.5 cm for three superficial layer thickness. The liquid phantom mixtures were made using a lipid-based liquid SMOFlipid (Fresenius Kabi), distilled water and glycerol to slow down the particle’s movement within the phantoms. The fast flow $\alpha D_{\text{B}}^{\text{fast}}$ is prepared without the glycerol and the model of the slow flow $\alpha D_{\text{B}}^{\text{slow}}$ is made using the 40% glycerol concentration. As the phantom absorption does not influence the dynamic of properties [27], no absorber was added to the solutions. To set the desired reduced scattering coefficient, various concentrations of the SMOFlipid were added into each mixture following the recipe in [28,29]. Moments of the measured DTOFs were used to calculate the absorption coefficient *μ*_a_ and the reduced scattering coefficient *μ*′_s_ [30]. The measured optical properties of all phantoms are *μ*_a_ = 0.003 mm^−1^ and *μ*′_s_ = 1 mm^−1^.

### 3.3 In vivo experiments protocol

In order to evaluate the performance of each SDS to recover the blood flow, we carry out series of stimulations including the post-occlusive reactive hyperaemia (PORH) test on healthy subject’s arm and brain cortex perfusion on the forehead of healthy adult volunteers during breath hold (BH) and hyperventilation (HV) tasks. The research protocol was approved by the bioethics committee at the military medical chamber in Warsaw, Poland (Approval No. 17/23).

The PORH test is a controllable in vivo stimulation to evaluate the performance of optical system to detect hemodynamic response and is commonly applied to compare different SDS ability to detect blood flow changes induced by occlusion [31–33]. This stimulation was carried out at each SDS separately on both left and right arms of four healthy volunteers (three females, one male, average age of 32). The optical probe was fixed to the forearm and the volunteers were sitting on a comfortable armchair. Data in each trial acquired using the following time scheme: 60 s of baseline followed by 40 s of arm occlusion (cuff pressure of 180 mmHg) and 60 s of release which was repeated twice per trial at each SDS separately. Thus, 16 data set for each SDS were collected.

BH and HV are systemic and not regional or localized stimulation that lead to cerebrovascular reactivity. They are routinely used to evaluate the ability of DCS systems to detect CBF changes [6,7,10,34–37]. In these tasks, the optical probe was located at the left side of the frontal lobe of the healthy volunteers following [6,38] and fixed using a plastic bandage approximately 1 cm above the eyebrow. The healthy volunteers (four females and two males with the average age of 36) were sitting on an armchair in a comfortable position (with body angle of 45^°^). The BH task consisted of a 60 s baseline, 30 s holding the breath without an initial inhale or exhale and 60 s recovery. The HV task, started with 60 s baseline, followed by 40 s attempt to fully exhale and inhale to maximize the respiratory exchange ratio and 60s recovery. Each task was repeated twice for every SDSs.

### 3.4 Data analysis

The *β* and $\alpha D_{\text{B}}$ are considered as two major measurands in the td-DCS technique. The *β* reflects the loss of spatial and temporal coherency of the detected light caused by moving particles. The td-DCS system parameter including laser coherence length, IRF width, number of modes in a detection fiber, as well as the width of the time gate affects this measurand [10,14,39]. The $\alpha D_{\text{B}}$ represents the concentration/fraction of moving particles times the particle diffuse coefficient [3,8,24].

#### Data quality

To achieve the best performance of the td-DCS at each SDS, we look for the optimal time gates to stratify the $\alpha D_{\text{B}}$ of superficial and deeper layers. To do that, we look into quality metrices based on the measurands of *β* and $\alpha D_{\text{B}}$ . The coefficient of variation *CV*_β_(*t*) is defined as the ratio of the standard deviation and the mean value of the *β*, calculated at a given time of flight *t*. The second quality metrics based on *β* measurand is the contrast to noise ratio *CNR*_β_(*t*) which evaluates the ratio of the dynamic range of the measured $g_{2}(t,\tau )$ and its standard deviation at short (τ→0) and long (τ→∞) delay times e.g. τ = 10^−6^ s and τ = 10^−2^ s [14,40]:

(5) $$CNR_{\beta }(t)=\;\frac{g_{2}(t,\tau \to 0)-g_{2}(t,\tau \to \infty )}{\sqrt{\sigma_{\tau \to 0}^{2}+\sigma_{\tau \to \infty \;\;}^{2}}}=\frac{\beta (t)}{\sqrt{\sigma_{\tau \to 0}^{2}+\sigma_{\tau \to \infty \;\;}^{2}}},$$

where $\sigma_{\tau \to 0}^{2}$ and $\sigma_{\tau \to \infty }^{2}$ are variances of the $g_{2}(t,\tau )$ at the short and long delay times *τ* respectively.

Consequently, we analyse the coefficient of variation of the flow $CV_{\alpha {\text{D}}_{\text{B}}}(t)$ at the given time of flight *t*. Further, the following contrast to noise ratio of the flow parameter $CNR_{\alpha {\text{D}}_{\text{B}}}(t)$ [39] is evaluated:

(6) $$CNR_{\alpha {\text{D}}_{\text{B}}}(t)=\;\frac{\alpha D_{\text{B}}(t)-\alpha D_{\text{B}}^{\text{slow}}(t)}{\sigma_{\alpha {\text{D}}_{\text{B}}}(t)},$$

where the $\alpha D_{\text{B}}(t)$ is the recovered flow (e.g. on a two-layers phantom), $\alpha D_{\text{B}}^{\text{slow}}(t)$ is the reference flow (slow) for 40% glycerol in the superficial layer (SL) and the $\sigma_{\alpha {\text{D}}_{\text{B}}}(t)$ is the standard deviation of the recovered flow at a given TOF *t*.

#### Time gating

In the td-DCS technique, selecting photons based on their TOF distinguishes short and long photon paths and stratifies the flow at different depth. However, the noise contribution increases for the late photons as the number of detected photons decreases exponentially with time. Although wide time gate can increase the photons count, it may mix photons penetrating at different depths. Here, we propose applying two time-gates to stratify the flow changes between superficial and deeper layers using the full width half maximum (FWHM) strategy. The FWHM of a DTOF is affected by the optical properties and SDS. In this approach the early gate starts at the FWHM on the rising edge of DTOF and ends on the maximum value. The late gate starts at the maximum and ends at the FWHM on the DTOF tail. Hence, keeps the balance between the sensitivity and the noise contribution.

#### Sensitivity metrics

To evaluate the performance of each SDS in stratifying the flow changes in superficial and deep layer, the sensitivity metrices were defined similarly to the [39] where the perturbation method was applied. These metrices are calculated at early and late time gates, giving the sensitivity to superficial and deep layer. Considering the phantoms geometry as in Fig. 2, the sensitivity to flow changes (perturbation) within the layers can be defined as:

(7) $$S(t)=\;\frac{[\alpha D_{\text{B}}(t)/\alpha D_{\text{B}}^{\text{slow}}(t)]-1}{[\alpha D_{\text{B}}^{\text{fast}}(t)/\alpha D_{\text{B}}^{\text{slow}}(t)]-1}\cdot 100\%=\frac{\alpha D_{\text{B}}(t)-\alpha D_{\text{B}}^{\text{slow}}(t)}{\alpha D_{\text{B}}^{\text{fast}}(t)-\alpha D_{\text{B}}^{\text{slow}}(t)}\cdot 100\%,$$

where *t* is the TOF, $\alpha D_{\text{B}}(t)$ is the recovered flow measured on the layered phantom, $\alpha D_{\text{B}}^{\text{slow}}(t)$ is the reference flow (40% glycerol) and $\alpha D_{\text{B}}^{\text{fast}}(t)$ is the ground truth perturbed flow (no glycerol). Therefore, Eq. (7) applied to the perturbation in the deep layer as shown in Fig. 2(b) will be marked as $S_{\text{DL}}(t)$ (depth sensitivity) and for the perturbation within the superficial layer (Fig. 2(c)) will be shown as $S_{\text{SL}}(t)$ (superficial sensitivity).

#### Beam expander effect

In this study, the used beam expander to shine the phantom/tissue increases the illumination area to 13 mm in diameter. Therefore, the actual SDS vary as 1.5 ± 0.65 cm, 2.0 ± 0.65 cm and 2.5 ± 0.65 cm. Although photon detection in these SDS ranges may affect the absolute $\alpha D_{\text{B}}$ , in td-DCS studies due to effects of laser coherence length, IRF width, gate start time and also gate width on this value, the flow changes is mostly presented by relative $\alpha D_{\text{B}}\;(r\alpha D_{\text{B}})$ [10,12]. Here in order to evaluate the impact of beam expander on $r\alpha D_{\text{B}}$ at each SDS, we defined the $r\alpha D_{\text{B}}=\alpha D_{\text{B}}^{\text{fast}}(t)/\alpha D_{\text{B}}^{\text{slow}}(t)$ and calculated this value on homogenous phantoms measured with and without the beam expander on the source fiber.

#### Statistical analysis

The Wilcoxon signed rank test was carried out with the p-values <0.05. For each presented boxplot, the central line notes the median, the bottom and top edges are the 25-th and 75-th percentiles respectively. The whiskers extend to the most extreme data points and the plus markers are considered outliers. Here, each data point for both phantom and in-vivo measurements was calculated with 2 s integration time which represents a trade-off between the long integration time to increase the signal to noise ratio and a sufficient temporal resolution to estimate dynamic flow changes.

## 4. Results

### 4.1 Phantom measurements

**Fig. 3.**
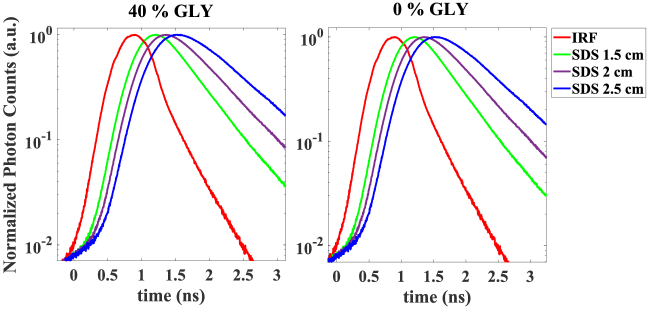
The IRF and DTOFs measured on homogenous phantoms (Fig. 2(a)) with 40% and no glycerol (GLY). The 0 time refers to 5% of the IRF rising edge.

In the first step, we have carried out measurements on homogeneous phantoms (Fig. 2(a)). The measured IRF and DTOFs are shown in Fig. 3. Increasing the SDS, shifts the DTOF to later time of flight and slightly broadens the curve. So, as the proposed time gating strategy is based on the FWHM, shifting the DTOF peak impacts the position of early and late gate at each SDS.

**Fig. 4.**
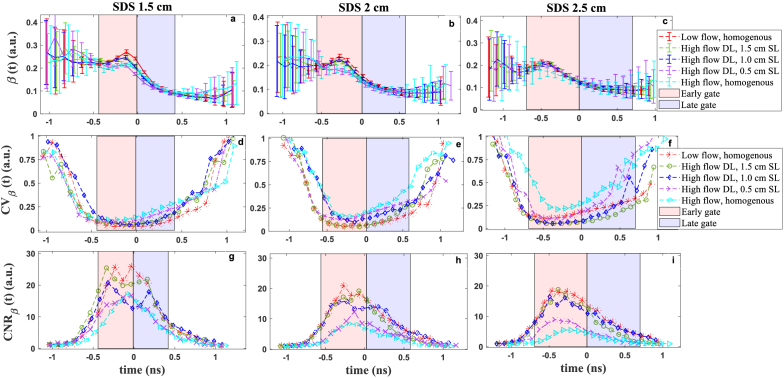
The *β* parameter quality metrics at different time of flight of photons *t* and SDSs. Time 0 is set at the maximum value of the DTOF. The calculated *β*(*t*) ((a)-(c)), coefficient of variation *CV*_β_(*t*) ((d)-(f)) and contrast to noise ratio *CNR*_β_(*t*) ((g)-(i)). Data points calculated within windows of 100 ps from 120 autocorrelation functions $g_{2}(t,\tau )$ collected with 2 s integration time each. Early and late gates set as FWHM of the DTOF. DL – deep layer, SL – superficial layer.

In order to identify the optimal DTOF range for time gating, we have calculated the metrices of *β*, $CV_{\beta }(t)$ and $CNR_{\beta }(t)$ (Fig. 4) for the time range of 2.2 ns around DTOFs peak measured during first and second steps of phantom experiment (Fig. 2(a), (b)). Here *β* has been calculated for every 100 ps of DTOF and reveal different trends over various time of flight.

As our system is equipped with the Vis-IR laser with relatively short coherence length (in comparison to continuous wave lasers), the photons lose more correlation at late time of flight leading to decreasing in *β*. The high *CV*_β_(t) in early and late time ranges follows the high standard deviation in *β*(*t*). The *CNR*_β_(*t*) depends on the ratio of *β* and noise of the measured autocorrelation $g_{2}(t,\tau )$ and follows the other metrics. According to these results, the time ranges with the highest *β* and *CNR*_β_ and the lowest *CV*_β_ are the optimal DTOF range for gating strategy which refers to DTOF peak. The early and late gate calculated by FWHM approach matches well with the optimal time ranges based on these measurand and metrics. Comparing the results from different SDSs, show increase in SDS leads to decrease in *β*(*t*), consequently increase the *CV*_β_(*t*) and decrease the *CNR*_β_(*t*). This is because at larger SDS, photons encounter more dynamic scattering events with losing more correlation.

**Fig. 5.**
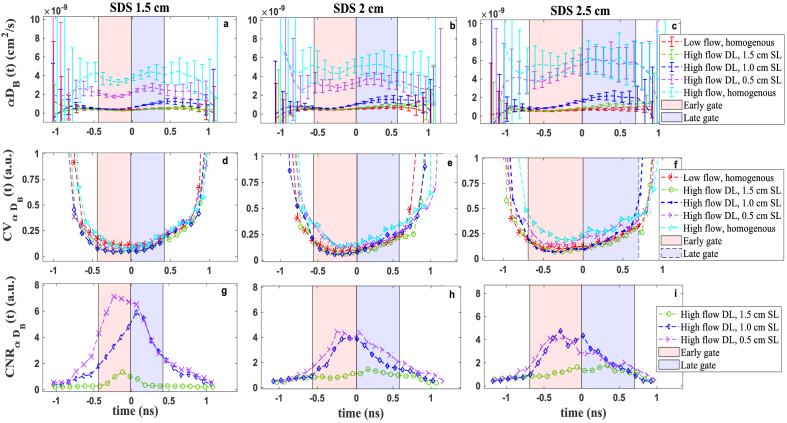
The *αD*_B_ parameter quality metrics at varying time of flight of photons and SDSs. Time 0 is set at the maximum of the DTOF. The recovered *αD*_B_(*t*) ((a)-(c)), coefficient of variation $CV_{\alpha {\text{D}}_{\text{B}}}(t)$ ((d)-(f)) and contrast to noise ratio $CNR_{\alpha {\text{D}}_{\text{B}}}(t)$ ((g)-(i)). Data point calculated within windows of 100 ps from 120 autocorrelation functions $g_{2}(t,\tau )$ collected with 2 s integration time each. Early and late gates set as FWHM gating approach. DL – deep layer, SL – superficial layer.

Figure 5(a)-(c) shows the $\alpha D_{\text{B}}(t)$ recovered for the corresponding *β* shown in Fig. 4(a)-(c). The recovered $\alpha D_{\text{B}}(t)$ for different superficial layer thicknesses, changes in the range between homogeneous phantoms of the superficial and deep layer. As the $\alpha D_{\text{B}}(t)$ refers to the speed of moving particles, is expected to be constant in homogenous medium. Although limited coherence of the laser leads to slightly differ the mean value of $aD_{B}$ along the various DTOF’s time range. According to our observation, lower $CV_{\alpha {\text{D}}_{\text{B}}}(t)$ calculated from the photons detected with the time ranges around DTOF peak reveals the lower error in calculation of $aD_{B}$ . Consequently, lower noise level in calculation of $aD_{B}$ for the photons detected within the DTOF peak, leads to significantly higher $CNR_{\alpha {\text{D}}_{\text{B}}}(t)$ . In contrast, the $CNR_{\alpha {\text{D}}_{\text{B}}}(t)$ for the photons detected within very short and very high time of flights is close to zero. As was shown for the *β*(*t*) and related metrics, the optimal DTOF range with lower quantification error and best recover quality metrics to calculate $\alpha D_{\text{B}}(t)$ is around the DTOF peak. The early and late time gates calculated with the proposed approach of the FWHM of DTOF follows well the optimal time ranges based on these metrics.

As was mentioned in description of Fig. 3, increasing the SDS leads to shift the DTOF peak to later time of flight and as a result gives rise to detect photons with longer pathlength coming from deeper layer. So, although lower *β* at longer SDS increases the standard deviation in calculation of $\alpha D_{\text{B}}(t)$ , higher number of photons detected from deep layer increase the differences between the recovered $\alpha D_{\text{B}}(t)$ and $\alpha D_{\text{B}}^{\text{slow}}(t)$ which leads to comparable $CNR_{\alpha {\text{D}}_{\text{B}}}$ especially for SDS 2 and 2.5 cm, see Fig. 5(g)-(i).

These metrics were also calculated for the phantom with high superficial flow, shown in Fig. 2(c). As the same optimal DTOF time ranges based on quality metrics were observed, the results are not shown.

**Fig. 6.**
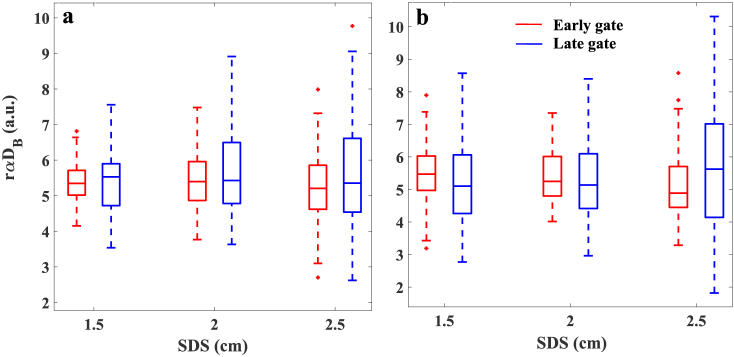
The $r\alpha D_{B}=\alpha D_{B}^{fast}(t)/\alpha D_{B}^{slow}(t)$ measured without the beam expander (a) and with the beam expander (b). Data calculated within early and late gates from 120 autocorrelation functions $g_{2}(t,\tau )$ collected with 2 s integration time each.

In order to evaluate the beam expander effect on detecting the relative changes caused by moving particles, we repeated the first step of measurements (Fig. 2(a)) without the beam expander at tip of laser fiber. Then we compared the results with data of first step measured by beam expander. Figure 6 presents the $raD_{B}$ calculated for the photons detected from early and late time gates at each SDS. According to these results no significant difference (p > 0.05) between $raD_{B}$ of early and late gates measured with and without beam expander can be observed at all SDSs.

**Fig. 7.**
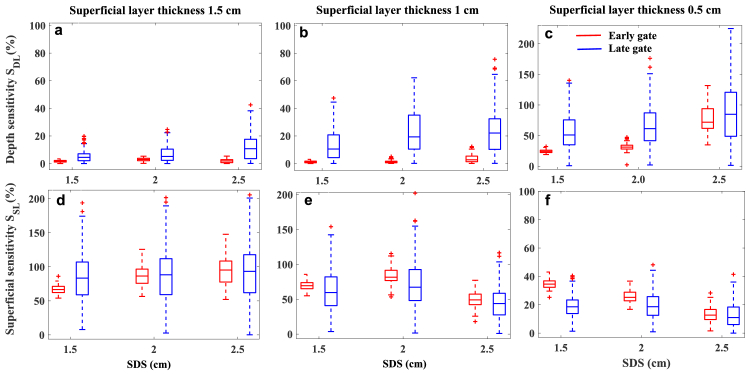
Sensitivity of the td-DCS to detect changes in deep ((a)-(c)) and superficial ((d)-(f)) layers at SDS of 1.5, 2 and 2.5 cm for various top layer thickness. Data calculated within early and late gates from 120 autocorrelation functions $g_{2}(t,\tau )$ collected with 2 s integration time each.

As in td-DCS, early and late gates are expected to carry information from superficial and deep layer, we evaluated each SDS sensitivity at defined gates to recover flow changes. Figure 7(a)-(c) shows the sensitivity of each SDS to detect flow in deep layer where the dynamics of particles in deep layer is more than superficial layer (second step measurement).

For all phantoms with different top layer thickness, higher SDS of the 2 and 2.5 cm improves the depth sensitivity (p-values <0.05) if the late gate is used. This is because, increasing SDS leads to shift the DTOF peak to later time and based on FWHM gating approach, increases the possibility of detecting photons with higher time of flight and longer pathlength. Although, this shift also affects the position of early gate and increase its depth sensitivity which is observed especially for phantom with low superficial layer thickness (0.5 cm). For phantoms with the top layer thicknesses of 1.5 and 1 cm (comparable to adult human grey mater depth), the difference between depth sensitivity of early and late gates is clearly visible which confirms the validity of our time gating approach and supports the expectation that early and late photons mostly are detected from superficial and deep layer respectively. As shown in Fig. 7(a), which represents a case closest to a human head measurement, the sensitivity of perfusion recovery within a deep (brain) layer located at 1.5 cm from the surface can increase on average 2.4 times by changing the SDS from 1.5 cm to 2.5 cm. This increase is visible only in the late gate as in this case the early gate does not show sensitivity to the deep layer.

Superficial sensitivity is another important parameter which has been calculated from third step phantom measurements with higher dynamics of particles in superficial layer. According to Fig. 7(d), in case of very thick top layer thickness (1.5 cm) both early and late gates at all SDSs are highly affected to changes in superficial layer. As a consequence, the results highlight that both defined early and late gate are prominent for monitoring changes in the superficial layer. This sensitivity is decreased for phantoms with lower top layer thickness (Fig. 7(e), (f)) and longer SDS.

### 4.2 In vivo measurements

**Fig. 8.**
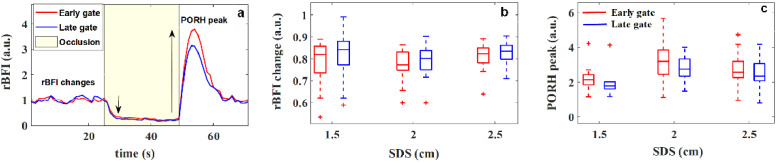
Hemodynamic changes in responses to the cuff occlusion test as measured at SDS = 2 cm. Representative haemodynamic response to the occlusion test registered on a healthy subject (a). The rBFI change (from baseline to the biological zero) (b) and the PORH peak (c) at three SDSs. The statistics ((b), (c)) are calculated from 16 datasets.

Cuff occlusion is a stimulation which allows to test the reactive hyperaemia of the forearm. During this test two hemodynamic responses can be observed: drop in the rBFI caused by occlusion (rBFI change in our study) and post-occlusive reactive hyperaemic (PORH), increase in the blood flow (PORH peak) (Fig. 8(a)). These parameters have been commonly used to study microvascular function in humans [31,33]. Although PORH is reproducible for one subject, its magnitude is heterogeneous across different subjects. Figure 8(b), (c) shows the results of the relative BFI (rBFI) changes and PORH tests across four subjects. The rBFI at the early and late time gate calculated normalizing the recovered flow by its baseline value.

According to our results, the rBFI changes (Fig. 8(b)) calculated from detected photons in early and late gates do not differ significantly within the measured subjects ( p≥0.05 ), regardless of the SDS. As the pressure induces on whole forearm, it affects skin and deeper muscles which leads to BFI changes detected from early and late gate. On the other hand, according to phantom experiments in case of low superficial layer thickness (here skin of forearm), the measured $aD_{B}$ of early gate at all SDSs is highly affected by dynamics of particles in deep layer. The differences between hyperaemia response is more noticeable for PORH peak at different SDS. As increase SDS leads to shift the DTOF peak to later time and based on FWHM gating approach, increase the possibility of detecting photon from deeper layer; the PORH peak derived from longer SDSs (2 and 2.5 cm) is stronger than for the short SDS ( p≤0.05 ). The stronger response might originate from the deep layer, the muscle [4].

**Fig. 9.**
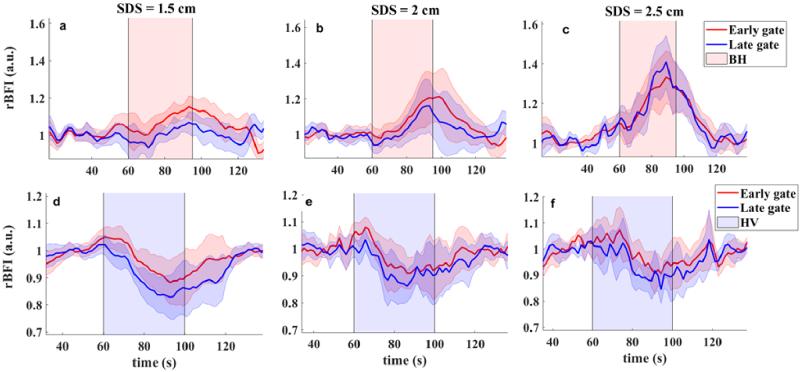
rBFI changes during BH (a)-(c) and HV (d)-(f) at three different SDSs. Solid line and shaded area refer to mean value and standard deviation of data calculated from 12 datasets at SDS = 1.5 and 2 cm, and 8 datasets at SDS = 2.5 cm (Data from two subjects at SDS 2.5 cm was collected with a significant noise level. This data was excluded from further averaging and statistical analysis). Shaded areas indicate the task period.

The breath hold (BH) and hyperventilation (HV) are expected to induce a global response in both extracerebral and brain tissues; however, most likely with different strength [41,42]. BH dilates the cerebral vasculature, increase the partial pressure of CO2 and as a result increases the blood flow in brain cortex [43,44]. The HV task is expected to decrease the arterial concentration of CO2 and inducing decrease in the brain blood flow [45,46]. Figure 9 shows the rBFI during the BH and HV tasks measured on healthy volunteers. Due to the hypercapnic hypoxia blood flow increases in response to the BH and slowly returns to baseline after release the breath. In the HV task, which causes hypocapnia, the blood flow decreases due to the vasoconstriction. These rBFI changes caused by breathing tasks can be observed at all SDSs. Same as cuff occlusion test, the rBFI at early and late gates was normalized by the baseline value.

**Fig. 10.**
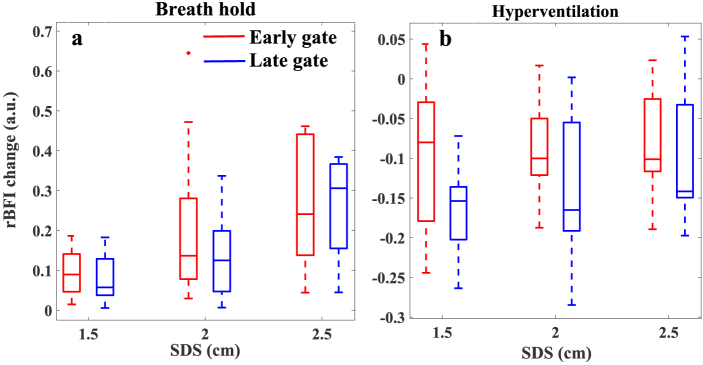
The statistical rBFI changes caused by BH (a) and HV (b) at three different SDSs. Each boxplot calculated from 12 datasets at SDS = 1.5 and 2 cm, and 8 datasets at SDS = 2.5 cm (Data from two subjects at SDS 2.5 cm was collected with a significant noise level and excluded from further averaging and statistical analysis). rBFI change refers to differences between the average rBFI of the baseline and the average rBFI in response to each task (from 10 seconds after the start time to 10 seconds after the completion of the task).

Our results show the blood flow changes in response to BH and HV can be observed in rBFI calculated from detected photons in both early and late gates. This is due to the fact that breathing tasks affect both superficial and deeper layer hemodynamic (scalp, skull, brain etc.) [5,47,48]. Referring to Fig. 7(d), for phantoms with the top layer thicknesses of 1.5 cm, both early and late gate are highly affected by dynamics of particles in superficial layer. However, for less top layer thickness of 1 cm (Fig. 7(e), (f)), the superficial sensitivity slightly decreases and the depth sensitivity shows the improvement for longer SDSs. Although these phantoms most likely describe the scalp and skull thickness of adults’ human brain, due to lack of information of the subjects’ head geometry, estimation the origin of the measured rBFI may not feasible. Taking advantage of the grand average of rBFI across subjects, enhances the statistical power and provides more general and clear results in response to these tasks. Figure 10(a) and (b) show the statistical analyses of rBFI changes caused by BH and HV respectively. The rBFI changes have been calculated by measuring the differences between the average rBFI of the baseline and the average rBFI in response to each task (from 10 seconds after the start time to 10 seconds after the completion of the task).

As shown in Fig. 10(a), the rBFI change induced by the BH increases at longer SDS, while it slightly decreases at longer SDSs for the HV test (Fig. 10(b)). The same trends in rBFI during the BH and HV have been reported using the cw-DCS technique [5,6], interferometric DCS [7] and td-DCS [10]. It might be due to stronger BFI changes in the superficial layer caused by the HV rather than BH which was also reported in [5]. According to Fig. 10, there is no significant difference between the rBFI at defined early and late gates at all SDSs. Also, only the statistical differences between rBFI change of BH at 1.5 and 2.5 cm are significant (p-values <0.05).

## 5. Discussion

In this study we evaluated the impact of SDS in td-DCS technique to recover the cerebral blood flow. To do that first we characterized the performance of td-DCS system at three different SDSs of 1.5, 2 and 2.5 cm using a series of measurements on homogenous and well-defined two-layered phantoms with various top layer thickness. Then we compared the BFI changes measured at large SDSs of 2 and 2.5 cm during occlusion test and breathing tasks and compared the results with what obtained at commonly SDS of 1.5 cm [9,10,23].

The quality metrics approach allowing to characterize the laser pulse shape and find the optimal DTOF ranges has been described in [14,39]. Here taking benefits of the combination of quality metrics based on the measurands of the *β* and $\alpha D_{\text{B}}$ , we showed a method to find the optimal time ranges in a td-DCS system to stratify dynamics of particles in superficial and deep layers. The time gating strategy is often applied using fixed gate start time and gate width for every subject with different optical properties of the tissues under investigation [9,10,49]. So, analysing early and late gates with the same temporal position for all subjects do not encounter the subject’s variability. Here, in the proposed gating strategy the gates positions and width are adjusted for every phantom/tissue. The FWHM gating approach, takes into account the SDS and the optical properties (*μ*_a_ and *μ*′_s_).

In order to evaluate the impact of the beam expander to stratify the relative dynamics changes as the product of td-DCS technique, the $r\alpha D_{\text{B}}$ was calculated for homogenous phantoms (40% and 0% glycerol concentration) measured with and without beam expander at the tip of the laser fiber. No significant difference was found between the $r\alpha D_{\text{B}}$ calculated for early and late time gate at each SDS. The influence of the beam expander diameter was also checked with the analytical solution of the autocorrelation field diffusion equation and for the semi-infinite medium geometry. The effect of varying SDS within the beam expander cancels out. The observable residuals at the *g*_1_ curve modelled for the centre of the beam expander and integrated within the beam expander do not exceed 1%.

Later using defined sensitivity metrices, the ability of different SDS, to detect dynamics changes of particles in the superficial and deep layers has been quantitatively compared. Results of the phantom measurements show that the thickness of the superficial layer has the most effect on the measured values of superficial sensitivity, deep sensitivity and recovered $\alpha D_{\text{B}}$ . Therefore, the large influence of the superficial layer thickness presents a significant obstacle in brain studies using the td-DCS, as the thickness of the extracerebral layer is usually unknown and varies greatly spatially between subjects [50]. In general, the system used in this research have limited capability to stratify the BFI changes in both superficial and deep layer. It has been reported that the desired FWHM of the laser IRF in the td-DCS is under 200 ps [23]. Therefore, the wide IRF (FWHM 550 ps) of the td-DCS system used in this research leads to mixing early and late photons at the measured DTOF.

As was mentioned before, BH and HV tasks are commonly used to evaluate DCS system ability to detect BFI changes which affect both skin and cerebral blood flow. Although higher rBFI changes caused by HV at short SDS [7] and BH at long SDS [35] has been reported which is in good agreement with our results; other stimulation with less effects on superficial layer is needed to evaluate td-DCS ability to stratify BFI changes from superficial and deeper layer.

Generally, the performance of td-DCS is not necessarily better compared to cw-DCS, mainly due to the relatively low photon flux in td-DCS since only a portion of the detected photons are selected [19].

## 6. Conclusions

In this study, we evaluated the performance of td-DCS at three different SDSs during series of phantom and in-vivo measurements. We used important measurands in td-DCS including *β* and *αD*_B_ and the related metrics to identify the DTOF time ranges for gating strategy. We found the optimal DTOF range with best recovery quality metrics and lower quantification error to calculate dynamics of particles is around the DTOF peak. Then we suggested an approach based on DTOF FWHM to define early and late time gates. By applying the sensitivity metrics we evaluated the ability of each SDS in defined early and late gate to detect dynamics of particles is superficial and deep layer. Finally, for each SDS the ability to detect blood flow changes during cuff occlusion and breathing tasks was tested. The rBFI changes caused by these tasks was detected at all SDSs. As BH and HV task have effects on both superficial and deeper layer, the same trend on rBFI changes was observed in early and late gate at all SDSs; although with different magnitude.

According to our results increasing the SDS leads to shift the DTOF peak to later time of flight and improvements the depth sensitivity. However, the overall performance of the td-DCS system is limited by the coherence length of the pulsed laser and IRF width. Therefore, using the time gating strategy without prior IRF correction may not be suitable for the td-DCS flow recovery. So, a robust method of the IRF influence consideration in the pipeline of processing the td-DCS data is needed.

## Data Availability

Data underlying the results presented in this paper may be obtained from the authors upon reasonable request.
